# Supplementary material for: Association of healthy lifestyle score with control of hypertension among treated and untreated hypertensive patients: a large cross-sectional study
Source: PeerJ. 2024 Apr 10;12:e17203. doi: 10.7717/peerj.17203 (PMC11015831; doi:10.7717/peerj.17203)
Supplement: Supplemental Information 2 [file peerj-12-17203-s002.docx]

STROBE Statement—checklist of items that should be included in reports of observational studies

|  | Item No. | Recommendation | Page  No. | Relevant text from manuscript |
| --- | --- | --- | --- | --- |
| **Title and abstract** | 1 | (*a*) Indicate the study’s design with a commonly used term in the title or the abstract | Page 1 | Association of healthy lifestyle score with control of hypertension among treated and untreated hypertensive patients: a large cross-sectional study |
|  |  | (*b*) Provide in the abstract an informative and balanced summary of what was done and what was found | Page 2 | Background. Hypertension is the largest single contributor to the global burden of mortality and disability. Evidence is limited regarding the association between combined healthy lifestyle score (HLS) and hypertension control among treated and untreated hypertensive patients. Therefore, we aimed to investigate the association between HLS and blood pressure control among treated and untreated hypertensive adults.  Methods. This cross-sectional study, including 311,994 hypertension patients, was conducted in Guangzhou using the data based on the Basic Public Health Services Projects in China. The HLS was defined by six low-risk lifestyle factors: healthy dietary habits, sufficient physical activity, ideal body mass index, suitable waist circumference, non-smoking, and no alcohol consumption. Controlled blood pressure was defined as systolic blood pressure <140 mmHg and diastolic blood pressure < 90 mmHg. A multivariable logistic regression model was used to estimate the association between the HLS and hypertension control after controlling for various confounders.  Results. HLS was found to be inversely associated with hypertension control among hypertensive patients. Compared with low HLS group (scored 0-2), the adjusted odds ratios (95% confidence intervals) of hypertension were 0.82 (0.79, 0.85), 0.66 (0.64, 0.68), 0.49 (0.48, 0.51), and 0.43 (0.41, 0.45) for the HLS scored 3, 4, 5, and 6 groups, respectively (*P*_trend_ <0.001). Hypertensive patients who adhered to a healthy lifestyle without taking medication had better blood pressure management than those who used medication and followed a healthy lifestyle.  Conclusion. HLS was associated with a reduced risk of uncontrolled blood pressure. |
| Introduction | | | |  |
| Background/rationale | 2 | Explain the scientific background and rationale for the investigation being reported | Page 3, 4 | Hypertension is the biggest single contributors to the burden of mortality and disability worldwide. From 1990 to 2019, the number of individuals with high blood pressure aged 30-79 doubled, with approximately 626 million females and 652 million males living with hypertension in 2019 worldwide. Around 75% of people with hypertension reside in low- and middle-income nation. The prevalence of hypertension among Chinese adults has also increased year by year with urbanization and aging of the population. Although managing hypertension is a national public health priority, the control rate of blood pressure remains low at 7.2% in China in 2014-1. It is important to identify effective strategies to manage hypertension.  Modifiable lifestyle factors, including dietary habits, physical activity, body mass index (BMI), waist circumference (WC), smoking, and alcohol drinking, have been separately shown to influence blood pressure levels. Studies suggested that a healthy diet, regular exercise, normal BMI and WC, and reducing alcohol consumption were beneficial for hypertension control. But there was a conflicting association between smoking status and blood pressure control. However, these studies have ignored the fact that lifestyle-related factors usually tend to coexist in individuals and together affect people's physical health. Thus, these lifestyle factors should be taken into account simultaneously, as well as their combined consequences.  Antihypertensive medication treatment is also an important part of hypertension management. However, using anti-hypertensive drugs may cause an increase in non-compliance with healthy lifestyles. So far, some studies have evaluated the relationship between joint lifestyle factors and hypertension control in treated hypertensive individuals. The relationship between lifestyle factors and blood pressure control have also been investigated in untreated hypertensive patient. However, few studies have simultaneously evaluated the association between lifestyle behavior and hypertension control among treated and untreated hypertensive patient, which could be an important step for blood pressure management. To our knowledge, only a cross-sectional study in Iran has investigated healthy lifestyle and hypertension control among those who aware and used antihypertensive medications and those who aware but not taking medication. But this study had a limited sample size and did not include people aged over 65 years. In addition, there are no related studies in China. |
| Objectives | 3 | State specific objectives, including any prespecified hypotheses | Page 4 | To fill these knowledge gaps, we aimed to investigate the association of a healthy lifestyle score (HLS), including dietary habits, physical activity, BMI, WC, smoking, and alcohol drinking with hypertension control among treated and untreated hypertensive patients in China. |
| Methods | | | |  |
| Study design | 4 | Present key elements of study design early in the paper | NA | NA |
| Setting | 5 | Describe the setting, locations, and relevant dates, including periods of recruitment, exposure, follow-up, and data collection | Page 4 | Hypertension patients who received the National Basic Public Health Services in Guangzhou in 2018 were recruited as the study subjects. The National Basic Public Health Services projects are the most basic healthcare service provided by the government free of charge to all residents in response to the main health problems existing in urban and rural residents, focusing on children, pregnant women, the elderly, and patients with chronic diseases. |
| Participants | 6 | (*a*) *Cohort study*—Give the eligibility criteria, and the sources and methods of selection of participants. Describe methods of follow-up  *Case-control study*—Give the eligibility criteria, and the sources and methods of case ascertainment and control selection. Give the rationale for the choice of cases and controls  *Cross-sectional study*—Give the eligibility criteria, and the sources and methods of selection of participants | Page 4, 5 | Hypertension patients who received the National Basic Public Health Services in Guangzhou in 2018 were recruited as the study subjects. A total of 375,912 hypertensive patients were included in this study. Inclusion criteria were as follows: aged ≥35 years, Guangdong natives or residents having lived in Guangzhou for at least half a year, with primary hypertension, and receiving physical examination. The following participants were excluded: repeat participants (n=13,284); participants who had missing or incomplete information on blood pressure and lifestyle factors (n =21,422); and participants with extreme values in blood pressure and lifestyle factors (n=29,212). Finally, 311,994 participants were included in the final data analysis. |
|  |  | (*b*) *Cohort study*—For matched studies, give matching criteria and number of exposed and unexposed  *Case-control study*—For matched studies, give matching criteria and the number of controls per case | NA | NA |
| Variables | 7 | Clearly define all outcomes, exposures, predictors, potential confounders, and effect modifiers. Give diagnostic criteria, if applicable | Page 6, 7 | Controlled hypertension was defined as mean SBP <140 mm Hg and mean DBP <90 mm Hg. We defined uncontrolled hypertension as mean SBP ≥140 mm Hg and/or mean DBP ≥90 mm Hg.  We derived an HLS based on information on six lifestyle factors—dietary habits, physical activity, BMI, WC, smoking, and alcohol drinking. Participants scored 1 point for each of the following protective lifestyle factors: healthy diet (balanced meat and vegetable and vegetarian-based), being physically active (exercise frequency ≥2 times weekly) (Yokokawa et al., 2014), optimal BMI (18.5-23.9 kg/m2), suitable WC (less than 90 cm for males and less than 85 cm for females), never smoking, and never drinking. These six scores were added together to give a final low risk lifestyle score ranging from 0 to 6, with lower scores indicating an unhealthier lifestyle. |
| Data sources/ measurement | 8* | For each variable of interest, give sources of data and details of methods of assessment (measurement). Describe comparability of assessment methods if there is more than one group | Page 5, 6 | All participants were face-to-face interviewed by trained medical staff at community-level health facilities using a structured questionnaire. The following information was collected: socio-demographic characteristics (age, sex, ethnicity, educational level, marital status), lifestyle factors (dietary habits, physical activity, smoking, and alcohol drinking), history of diabetes, family history of hypertension, and medication information. Dietary habits were divided into balanced meat and vegetable, meat-based, vegetarian-based, salt-loving, oil-loving, and sugar-loving. Physical activity levels were assessed by exercise frequency. The frequency of physical activity was categorized as never, occasionally, more than once a week, and daily, where occasionally and more than once a week were calculated based on once a week and three times a week, respectively. Smoking status was divided into never smokers and smokers (former and current smoking). We defined never smokers as participants who reported never smoking, smokers as participants who reported current smoking and ever smokers. Similarly, drinking status was segmented into never drinkers and drinkers (ever and current drinking). Never drinkers were defined as participants who reported never drinking, drinkers as participants who reported current drinkers and former drinkers.  Standardized methods were used to measure participants' height, weight, and WC. BMI was calculated by dividing body weight (in kilograms) by the square of height (in meters).  Systolic and diastolic blood pressure was measured on the seating position in both arms. The mean value of the two measurements from left and right arms was considered as person’s blood pressure measurements. |
| Bias | 9 | Describe any efforts to address potential sources of bias | Page 7 | The multivariable model was adjusted for potential confounders, which were selected based on the disparities by comparing the baseline characteristics of the controlled with the uncontrolled groups. The following variables were adjusted for in the multivariable models: age, sex, ethnicity, educational level (primary school or below, junior high school, senior high school, college or above and unknown), marital status, history of diabetes, family history of hypertension, and antihypertensive drugs use. |
| Study size | 10 | Explain how the study size was arrived at | NA | NA |

Continued on next page

| Quantitative variables | 11 | Explain how quantitative variables were handled in the analyses. If applicable, describe which groupings were chosen and why | Page 6, 7 | According to the Working Group on Obesity in China, underweight, normal weight, overweight, and obesity were defined as BMI <18.5, 18.5–23.9, 24.0–27.9, and ≥28.0 kg/m2, respectively. Continuous variables are expressed as the mean ± standard deviation (SD) and categorical variables are expressed as numbers (percentages). |
| --- | --- | --- | --- | --- |
| Statistical methods | 12 | (*a*) Describe all statistical methods, including those used to control for confounding | Page 7, 8 | We categorized the HLS into five groups (0-2, 3, 4, 5, and 6). Since the number of participants with HLS scored 0 and 1 was very small (n = 158 and 2938, respectively), we merged the three lowest categories and considered those with HLS of 0-2 as the reference category in all analyses. Continuous variables are expressed as the mean ± standard deviation (SD) and categorical variables are expressed as numbers (percentages). Comparisons of basic characteristics of controlled and uncontrolled hypertension were evaluated using chi-square tests (for categorical variables) and t-tests (for continuous variables). Multivariate logistic regression model was used to estimate odds ratio (OR) and 95% confidence intervals (CI) for the analysis of the relationship between HLS and control of hypertension, considering participants following an unhealthy lifestyle as the reference. The multivariable model was adjusted for potential confounders, which were selected based on the disparities by comparing the baseline characteristics of the controlled with the uncontrolled groups. The following variables were adjusted for in the multivariable models: age, sex, ethnicity, educational level (primary school or below, junior high school, senior high school, college or above and unknown), marital status, history of diabetes, family history of hypertension, and antihypertensive drugs use.  All statistical analysis were performed using R x64 4.1.1. Statistical significance was determined at a two tailed P value of less than 0.05. |
|  |  | (*b*) Describe any methods used to examine subgroups and interactions | Page 7 | Stratified analyses were conducted by age (<65 vs ≥65 years), sex (males vs females), and antihypertensive drugs use (without taking antihypertensive medication vs taking antihypertensive medication). The interactive effects were calculated by including interaction terms in the multiple regression model. To determine the association of each component of the HLS with hypertension control, we constructed univariate adjustment models controlling for the above covariates, as well as the other components of the HLS for each component. |
|  |  | (*c*) Explain how missing data were addressed | Page 5 | The following participants were excluded: participants who had missing or incomplete information on blood pressure and lifestyle factors (n =21,422); and participants with extreme values in blood pressure and lifestyle factors (n=29,212). |
|  |  | (*d*) *Cohort study*—If applicable, explain how loss to follow-up was addressed  *Case-control study*—If applicable, explain how matching of cases and controls was addressed  *Cross-sectional study*—If applicable, describe analytical methods taking account of sampling strategy | NA | NA |
|  |  | (*e*) Describe any sensitivity analyses | NA | NA |
| Results | | | | |
| Participants | 13* | (a) Report numbers of individuals at each stage of study—eg numbers potentially eligible, examined for eligibility, confirmed eligible, included in the study, completing follow-up, and analysed | Page 4, 5 | A total of 375,912 hypertensive patients were included in this study. Inclusion criteria were as follows: aged ≥35 years, Guangdong natives or residents having lived in Guangzhou for at least half a year, with primary hypertension, and receiving physical examination. The following participants were excluded: repeat participants (n=13,284); participants who had missing or incomplete information on blood pressure and lifestyle factors (n =21,422); and participants with extreme values in blood pressure and lifestyle factors (n=29,212). Finally, 311,994 participants were included in the final data analysis. |
|  |  | (b) Give reasons for non-participation at each stage | NA | NA |
|  |  | (c) Consider use of a flow diagram | Page 5 | Finally, 311,994 participants were included in the final data analysis (Fig. 1). |
| Descriptive data | 14* | (a) Give characteristics of study participants (eg demographic, clinical, social) and information on exposures and potential confounders | Page 8 | As shown in Table 1, of the 311,994 participants, 243,101 (77.92%) were 65 years and older, 124,116 (39.78%) were males; 187,369 (60.06%) had controlled hypertension, and 256,328 (82.16%) used antihypertensive medication. Compared with participants who had controlled blood pressure, those with uncontrolled hypertension were more likely to be older, had higher proportions of females, be less educated, and less married. Participants with uncontrolled hypertension were also more likely to have a higher BMI and WC, less physical activity, unhealthy dietary habits, be smokers and drinkers, have lower HLS, less history of diabetes, and less antihypertensive medication use than controlled hypertensive patients. There was no significant difference in ethnicity (*P* = 0.361), and family history of hypertension (*P* = 0.323). |
|  |  | (b) Indicate number of participants with missing data for each variable of interest | NA | NA |
|  |  | (c) *Cohort study*—Summarise follow-up time (eg, average and total amount) | NA | NA |
| Outcome data | 15* | *Cohort study*—Report numbers of outcome events or summary measures over time | NA | NA |
|  |  | *Case-control study—*Report numbers in each exposure category, or summary measures of exposure | NA | NA |
|  |  | *Cross-sectional study—*Report numbers of outcome events or summary measures | Page 8 | As shown in Table 1, of the 311,994 participants, 187,369 (60.06%) had controlled hypertension, |
| Main results | 16 | (*a*) Give unadjusted estimates and, if applicable, confounder-adjusted estimates and their precision (eg, 95% confidence interval). Make clear which confounders were adjusted for and why they were included | Page 7, 8 | The multivariable model was adjusted for potential confounders, which were selected based on the disparities by comparing the baseline characteristics of the controlled with the uncontrolled groups. The following variables were adjusted for in the multivariable models: age, sex, ethnicity, educational level (primary school or below, junior high school, senior high school, college or above and unknown), marital status, history of diabetes, family history of hypertension, and antihypertensive drugs use.  Table 2 shows the association of HLS with hypertension control. Compared with patients with the HLS scored 0-2, the multivariable adjusted ORs and 95% CIs for those with a score of 3, 4, 5 and 6 was 0.82 (0.79, 0.85), 0.66 (0.64, 0.68), 0.49 (0.48, 0.51), and 0.43 (0.41, 0.45; *P*_trend_ < 0.001), respectively. |
|  |  | (*b*) Report category boundaries when continuous variables were categorized | Page 7 | We categorized the HLS into five groups (0-2, 3, 4, 5, and 6). Since the number of participants with HLS scored 0 and 1 was very small (n = 158 and 2938, respectively), we merged the three lowest categories and considered those with HLS of 0-2 as the reference category in all analyses. |
|  |  | (*c*) If relevant, consider translating estimates of relative risk into absolute risk for a meaningful time period | NA | NA |

Continued on next page

| Other analyses | 17 | Report other analyses done—eg analyses of subgroups and interactions, and sensitivity analyses | Page 8, 9 | Stratified analysis by age showed that the inverse association between HLS and hypertension control was stronger in younger (< 65 years) than in older (≥ 65 years) participants (*P*_interaction_ < 0.001). The adjusted ORs for the highest HLS relative to the HLS scored 0-2 were 0.30 (95% CI 0.28,0.33; *P*_trend_ < 0.001) for participants younger than 65 years and 0.47 (95% CI 0.45,0.50; *P*_trend_ < 0.001) for participants equal to and older than 65 years, respectively. A sex-stratified analysis showed that the negative association between HLS and blood pressure control was more apparent in males than in females (*P*_interaction_ = 0.016). Compared with the HLS scored 0-2, adjusted ORs for the highest HLS were 0.38 (95% CI 0.36, 0.40, *P*_trend_ <0.001) for males and 0.56 (95% CI 0.50, 0.62; *P*_trend_ <0.001) for females, respectively. Additionally, the inverse association between HLS and hypertension control was more evident in hypertensive patients without medication use than in those with medication use (*P*_interaction_ < 0.001). Compared the highest (HLS scored 6) with the lowest HLS (HLS scored 0-2), an adjusted OR (95% CIs) was 0.36 (0.33, 0.39, *P*_trend_ <0.001) in people who used antihypertensive medication and 0.45 (0.43, 0.46, *P*_trend_ <0.001) in those who did not use any antihypertensive medication (Table 2).  The associations between individual lifestyle factors and HLS and controlled hypertension are described in Fig. 2 and Fig. 3. Dietary habits, physical activity, BMI, WC, smoking, and alcohol consumption were independently negatively associated with blood pressure control. Moreover, those who adopted all six healthy lifestyle factors had better blood pressure control than those who adopted only single factor. Similarly, subgroup analysis by age, sex, and antihypertensive drugs use showed that healthy dietary habits, active physical activity, ideal BMI and WC, and non-drinking were associated with well-controlled blood pressure. As for smoking status, we found that smoking was inversely associated with hypertension control in males, but there was an unexpected positive relationship between non-smoking and controlled hypertension in females. |
| --- | --- | --- | --- | --- |
| Discussion | | | | |
| Key results | 18 | Summarise key results with reference to study objectives | Page 10 | The findings showed that HLS was inversely associated with hypertension control among hypertensive patients. Hypertensive patients who followed a healthy lifestyle without taking medication had better blood pressure management than those who used medication and followed a healthy lifestyle. |
| Limitations | 19 | Discuss limitations of the study, taking into account sources of potential bias or imprecision. Discuss both direction and magnitude of any potential bias | Page 14, 15 | Nevertheless, there are also several limitations to this study. First, the recall bias cannot be excluded because of self-reported lifestyle factors. However, we provided professional training to health workers to reduce this bias as much as possible. Second, the definition of a healthy diet was only assessed based on simple dietary habits due to the lack of data on nutrient intake. We could not evaluate the intake of various nutrients in the model, especially sodium, which has a significant impact on blood pressure. Therefore, our definition of a healthy diet was constrained to our study population and there may be restrictions to extrapolation of results. However, healthy eating factor in a cohort study in Japan was also defined based on dietary habits. Third, reverse-causality bias is inherent in cross-sectional analyses, preventing the confirmation of a causal association between healthy lifestyle and controlled hypertension. Fourth, we controlled for confounding bias by adjusting the multivariate mixture model and stratified analysis, but the residual confounders could not be completely excluded owning to the observational study design. Fifth, we define controlled blood pressure by one measurement rather than the average of multiple measurements. However, we measure the blood pressure of the left and right arms and take their average as the blood pressure value. Lastly, since our study was a cross-sectional study, information on lifestyle and medications was collected only once at baseline. We could not investigate the relationship between changes in lifestyle and medication utilization and blood pressure control. Therefore, prospective studies are needed to further explore the relationship between lifestyle and hypertension control. |
| Interpretation | 20 | Give a cautious overall interpretation of results considering objectives, limitations, multiplicity of analyses, results from similar studies, and other relevant evidence | Page 10-15 | We identified a strong inverse association between HLS and hypertension control. Consistent with our results, Akbarpour et al found that the risk of uncontrolled hypertension in people with an unhealthy lifestyle was approximately 37% more than that in people who adhered to moderate lifestyle. A cross-sectional study in France found that modifiable unhealthy lifestyle factors were associated with an increased risk of uncontrolled hypertension only in treated hypertensive subjects. The FRESH study in Japan reported that maintaining a healthy lifestyle was a protective factor for blood pressure management. A Chinese Community Intervention Trial found that the rate of blood pressure control improved by 56.1% after lifestyle intervention over 1 year. Moreover, in the PREMIER clinical trial of 810 participants with nonoptimal blood pressure, a 6-month lifestyle intervention of weight loss, sodium restriction, physical activity enhancement, alcohol intake restriction, and improved diet quality significantly reduced SBP by 4.3 mmHg. Our findings are in line with earlier research. We found that a higher HLS was associated with a lower risk of uncontrolled hypertension, suggesting the important role of lifestyle factors on management of hypertension. Considering the combined evidence, formulation and application of effective lifestyle intervention strategies are expected to improve hypertension management.  Our study showed that 82.16% hypertensive patients took antihypertensive medication. Patients who used medication and adhered to a healthy lifestyle had worse blood pressure control than those who adhered to a healthy lifestyle and without medication. There are some possible explanations for the poorer blood pressure control in people who adhered to a healthy lifestyle and used antihypertensive drugs. First, the prevalence of diabetes and family history of hypertension was higher in hypertensive patients who used medication than those who did not use medication in our study. Second, people who do not take medication may have a lower level of blood pressure than those who are taking medication. Lastly, uncontrolled hypertension in people taking medication may be due to lower adherence. To our knowledge, only one study has simultaneously examined the relationship between HLS and blood pressure control among treated and untreated hypertensive patients. A cross-sectional study in Iran including 2577 participants with hypertension found that the risk of uncontrolled hypertension in people with good lifestyle behaviors was 1% and 45% lower than those with poor lifestyle behaviors among treated and untreated hypertensive patients, respectively. However, the study's findings were not statistically significant, which may be because of their relatively small sample size. In addition, the Dongfeng-Tongji cohort study in China showed that patients who adhered to a healthy lifestyle but did not use medications had a lower mortality rate than patients who used drugs and adhered to a healthy lifestyle. This suggested that when managing patients with hypertension, health care workers need to place a greater emphasis on maintaining a healthy lifestyle, especially among those whose blood pressure is still poorly controlled by using antihypertensive drugs.  Our study showed that the inverse association between HLS and hypertension control was more evident in younger patients than in older ones. It may be due to the fact that the elderly are more likely to accompany by other complications, such as coronary and peripheral atherosclerosis, renal dysfunction, and diabetes, therefore, it is more difficult to control blood pressure through a healthy lifestyle among the elderly. We found that the negative association between HLS and control of hypertension was more apparent in males than in females. This finding is similar to the result of a cross-sectional study in France. The potential reason might be that females had higher prevalence of diabetes and family history of hypertension compared to males. Adopting a healthy lifestyle is important to control blood pressure, especially among older and female patients.  The analysis investigating blood pressure control in relation to individual lifestyle factors suggested that healthy diet, active physical activity, optimal BMI and WC, and non-drinking were independently inversely correlated with uncontrolled hypertension. These findings are similar to those of previous studies that examined the associations between individual lifestyle factor and hypertension control. The present study adds to the evidence that healthy diet, physical activity, ideal BMI and WC, and never smoking contributes to the management of blood pressure. Our findings also showed that those who adopted all six healthy lifestyle factors had better blood pressure control than those who adopted only single lifestyle factor. A study involving 1018 Irish adults showed that those with four and more protective lifestyles had a lower risk of developing hypertension than those with a single lifestyle. The Aerobics Center Longitudinal Study found that those with only a single lifestyle were more likely to develop hypertension than those with five healthy lifestyles. This study, as well as previous studies, indicates that the HLS is a combined score that shows the extent to which an individual leads a healthy lifestyle overall.  The relationship between smoking and blood pressure control is controversial. Niu et al observed that smoking was significantly associated with higher blood pressure levels. We found that smoking was negatively associated with uncontrolled hypertension in males. While in females, surprisingly, the result showed a positive association between smoking and hypertension control. Similar to our study, Cherfan et al found that current smoking was associated with increased prevalence of controlled hypertension. The reasons for the paradoxical and opposite relationship between smoking and uncontrolled blood pressure are elusive and deserve further study. Nevertheless, a large amount of evidence showed that smoking was an essential risk factors of hypertension. Therefore, health workers should still provide smoking cessation advice to all people with hypertension.  To our knowledge, no study has examined the association of WC in combination with other lifestyle factors and blood pressure control. Our study extends previous findings with a comprehensive definition of HLS to assess the association of six lifestyle factors including diet, exercise, BMI, WC, drinking and alcohol consumption, with control of hypertension. Evidence regarding the association of WC in combination with other lifestyle factors with other diseases is also limited. The China Kadoorie Biobank and UK Biobank study found that among participants with high genetic risk of childhood obesity, maintaining a healthy lifestyle (currently non-smoking, moderate alcohol consumption, regular physical activity, healthy diet, appropriate BMI and WC) was associated with a 69% and 80% lower risk of type 2 diabetes compared with those with an unhealthy lifestyle. A large study in the UK found that a good lifestyle including diet, physical activity, smoking, alcohol consumption, BMI, and WC is correlated with a 31% reduced risk of invasive breast cancer in females after menopause.  Our study has certain strengths. First, the main advantage of this study is that it was undertaken on a large sample population of Chinese adults with hypertension. Next, we examined the relationship between more comprehensive HLS, including dietary habits, physical activity, BMI, WC, smoking, and alcohol drinking, and blood pressure control. Moreover, the relationship between HLS and blood pressure control was assessed among treated and untreated hypertensive patients.  Nevertheless, there are also several limitations to this study. First, the recall bias cannot be excluded because of self-reported lifestyle factors. However, we provided professional training to health workers to reduce this bias as much as possible. Second, the definition of a healthy diet was only assessed based on simple dietary habits due to the lack of data on nutrient intake. We could not evaluate the intake of various nutrients in the model, especially sodium, which has a significant impact on blood pressure. Therefore, our definition of a healthy diet was constrained to our study population and there may be restrictions to extrapolation of results. However, healthy eating factor in a cohort study in Japan was also defined based on dietary habits. Third, reverse-causality bias is inherent in cross-sectional analyses, preventing the confirmation of a causal association between healthy lifestyle and controlled hypertension. Fourth, we controlled for confounding bias by adjusting the multivariate mixture model and stratified analysis, but the residual confounders could not be completely excluded owning to the observational study design. Fifth, we define controlled blood pressure by one measurement rather than the average of multiple measurements. However, we measure the blood pressure of the left and right arms and take their average as the blood pressure value. Lastly, since our study was a cross-sectional study, information on lifestyle and medications was collected only once at baseline. We could not investigate the relationship between changes in lifestyle and medication utilization and blood pressure control. Therefore, prospective studies are needed to further explore the relationship between lifestyle and hypertension control. |
| Generalisability | 21 | Discuss the generalisability (external validity) of the study results | Page 14 | Therefore, our definition of a healthy diet was constrained to our study population and there may be restrictions to extrapolation of results. However, healthy eating factor in a cohort study in Japan was also defined based on dietary habits. |
| Other information | |  | | |
| Funding | 22 | Give the source of funding and the role of the funders for the present study and, if applicable, for the original study on which the present article is based | Page 16 | The authors received no funding for this work. |

*Give information separately for cases and controls in case-control studies and, if applicable, for exposed and unexposed groups in cohort and cross-sectional studies.

**Note:** An Explanation and Elaboration article discusses each checklist item and gives methodological background and published examples of transparent reporting. The STROBE checklist is best used in conjunction with this article (freely available on the Web sites of PLoS Medicine at http://www.plosmedicine.org/, Annals of Internal Medicine at http://www.annals.org/, and Epidemiology at http://www.epidem.com/). Information on the STROBE Initiative is available at www.strobe-statement.org.
