# Supplementary material for: Association of healthy lifestyle score with control of hypertension among treated and untreated hypertensive patients: a large cross-sectional study
Source: PeerJ. 2024 Apr 10;12:e17203. doi: 10.7717/peerj.17203 (PMC11015831; doi:10.7717/peerj.17203)
Supplement: Supplemental Information 3 [file peerj-12-17203-s003.docx]

**Table S1** Baseline characteristics for participants included and excluded

| **Characteristics** | **Included, No. (%)** | **Excluded, No. (%)** | ***P* value** |
| --- | --- | --- | --- |
| Overall | 311994 | 50634 |  |
| Age, years |  |  | <0.001 |
| <65 | 68893 (22.08) | 12623 (24.93) |  |
| ≥65 | 243101 (77.92) | 38011 (75.07) |  |
| Sex |  |  | <0.001 |
| Males | 124116 (39.78) | 23030 (45.48) |  |
| Females | 187878 (60.22) | 27604 (54.52) |  |
| Ethnicity |  |  | 0.229 |
| Han | 311497 (99.84) | 50541 (99.82) |  |
| Others | 497 (0.16) | 93 (0.18) |  |
| Educational level |  |  | <0.001 |
| Primary school or below | 92067 (29.51) | 15278 (30.17) |  |
| Junior high school | 63277 (20.28) | 10868 (21.46) |  |
| Senior high school/Secondary technical school | 69056 (22.13) | 10864 (21.46) |  |
| College or above | 85857 (27.52) | 13299 (26.26) |  |
| Unknown | 1737 (0.56) | 325 (0.64) |  |
| Marital status |  |  | 0.971 |
| Married | 275026 (88.15) | 44631 (88.14) |  |
| Others | 36968 (11.85) | 6003 (11.86) |  |
