# Supplementary material for: Association of healthy lifestyle score with control of hypertension among treated and untreated hypertensive patients: a large cross-sectional study
Source: PeerJ. 2024 Apr 10;12:e17203. doi: 10.7717/peerj.17203 (PMC11015831; doi:10.7717/peerj.17203)
Supplement: Supplemental Information 4 [file peerj-12-17203-s004.pdf]

"Informed Consent Waiver" Application

Ethical Review Committee for Biomedical Research, School of Public Health, Sun Yat-sen University:

I am conducting a research project titled "Study on the Blood Pressure and Blood Glucose Control of Hypertensive and Diabetic Patients and Its Influencing Factors". This research primarily involves the extraction of secondary data from the Guangzhou National Basic Public Health Service database concerning hypertensive and diabetic patients. This study does not directly involve patients. Therefore, I hereby request an exemption from providing informed consent forms.

Principal Investigator: Zhang Caixia

December 23, 2022
